# Increased Clearance of Reactive Aldehydes and Damaged Proteins in Hypertension-Induced Compensated Cardiac Hypertrophy: Impact of Exercise Training

**DOI:** 10.1155/2015/464195

**Published:** 2015-04-14

**Authors:** Juliane Cruz Campos, Tiago Fernandes, Luiz Roberto Grassmann Bechara, Nathalie Alves da Paixão, Patricia Chakur Brum, Edilamar Menezes de Oliveira, Julio Cesar Batista Ferreira

**Affiliations:** ^1^Department of Anatomy, Institute of Biomedical Sciences, University of Sao Paulo, 05508-000 Sao Paulo, SP, Brazil; ^2^School of Physical Education and Sport, University of Sao Paulo, 05508-030 Sao Paulo, SP, Brazil

## Abstract

*Background*. We previously reported that exercise training (ET) facilitates the clearance of damaged proteins in heart failure. Here, we characterized the impact of ET on cardiac protein quality control during compensated ventricular hypertrophy in spontaneously hypertensive rats (SHR). *Methods and Results*. SHR were randomly assigned into sedentary and swimming-trained groups. Sedentary SHR displayed cardiac hypertrophy with preserved ventricular function compared to normotensive rats, characterizing a compensated cardiac hypertrophy. Hypertensive rats presented signs of cardiac oxidative stress, depicted by increased lipid peroxidation. However, these changes were not followed by accumulation of lipid peroxidation-generated reactive aldehydes and damaged proteins. This scenario was explained, at least in part, by the increased catalytic activity of both aldehyde dehydrogenase 2 (ALDH2) and proteasome. Of interest, ET exacerbated cardiac hypertrophy, improved ventricular function, induced resting bradycardia, and decreased blood pressure in SHR. These changes were accompanied by reduced cardiac oxidative stress and a consequent decrease in ALDH2 and proteasome activities, without affecting small chaperones levels and apoptosis in SHR. *Conclusion*. Increased cardiac ALDH2 and proteasomal activities counteract the deleterious effect of excessive oxidative stress in hypertension-induced compensated cardiac hypertrophy in rats. ET has a positive effect in reducing cardiac oxidative stress without affecting protein quality control.

## 1. Introduction

Hypertension affects 1.5 billion people worldwide and costs annually ~1 trillion dollars [1, 2]. Hypertension-induced cardiac hypertrophy represents a compensatory adaptation in response to pressure overload and results in preserved ventricular function at early stages. However, sustained pressure overload lately results in pathological cardiac remodeling and ventricular dysfunction and ultimately leads to heart failure [3]. Therefore, blocking the transition from early-stage cardiac hypertrophy to decompensated ventricular remodeling is a crucial step to reduce hypertension-related morbidity and mortality [4, 5].

Despite the growing knowledge regarding the molecular basis of hypertension pathophysiology, little is known about the protein quality control profile in hypertension-induced cardiac hypertrophy. The protein quality control plays a central role in the maintenance of cardiac homeostasis by detecting, repairing, and disposing cytotoxic damaged proteins [6]. We have previously demonstrated that pharmacological activation of protein quality control-related machinery protects against cardiac ischemia-reperfusion injury and heart failure in rodents [7, 8]. More recently, we showed that aldehyde dehydrogenase 2 (ALDH2), a mitochondrial enzyme responsible for the detoxification of reactive aldehydes, plays a key role in protecting against cardiovascular diseases by counteracting the accumulation of aldehyde-induced damaged protein [9–11]. Indeed, accumulation of reactive aldehydes such as 4-hydroxynonenal (4-HNE) impairs cardiac protein quality control by disrupting ubiquitin proteasome system (UPS) [12].

Aerobic exercise training is an important nonpharmacological strategy for preventing and treating cardiovascular diseases [13, 14]. We have recently reported that exercise training reverses pathological cardiac remodeling in hypertensive rats [15]. Of interest, exercise training also improves the clearance of damaged proteins in heart failure [16], which highlights its role in regulating cardiac protein quality control. However, the mechanisms underlying the exercise-mediated benefits in hypertension are poorly understood. In the present study, we demonstrated a disrupted redox balance and a compensatory activation of proteasome and ALDH2 during compensated cardiac hypertrophy in SHR. Moreover, exercise training had a positive effect in reducing cardiac oxidative stress without affecting protein quality control in hypertension.

## 2. Methods

### 2.1. Animals and Study Design

Male spontaneously hypertensive rats (SHR) from 12 to 22 weeks of age were used as a model of compensated cardiac hypertrophy. Wistar Kyoto rats were used as controls. At 12 weeks of age SHR were randomly assigned into sedentary (SHR) and swimming-trained (SHRt) groups. Rats were maintained in a 12:12 h light-dark cycle and temperature-controlled environment (22°C) with free access to standard laboratory chow (Nuvital Nutrientes, Curitiba, PR, Brazil) and tap water. This study was conducted in accordance with the ethical principles in animal research adopted by the Brazilian College of Animal Experimentation (http://www.cobea.org.br/). The animal care and protocols in this study were reviewed and approved by the Ethical Committee of Medical School at University of São Paulo (2007/35).

### 2.2. Swimming Training Protocol

SHR performed swimming training over ten weeks, 5 days/week, 60 min/day, in an apparatus adapted for rats containing warmed water (30–32°C), as described elsewhere [15, 17]. The swimming training duration and workload were progressively increased until the rats could swim for 60 min/day wearing caudal dumbbells weighing 5% of their body weight. Thereafter, duration and workload were constant. Untrained and control rats were placed in the swimming apparatus for 10 minutes twice a week without workload to mimic the water stress associated with the experimental protocol.

### 2.3. Measurement of Aerobic Capacity

Exercise capacity, estimated by total distance run, was evaluated using a graded treadmill exercise protocol as previously described [18]. Rats were submitted to exercise testing before and after swimming training protocol. Briefly, after being adapted to treadmill over 5 days (10 min each session), rats ran on a treadmill until exhaustion. Exercise intensity started at 6 m·min^−1^ and was increased by 3 m·min^−1^ every 3 min. The same protocol was used to measure peak oxygen uptake (VO_2_). Animals were placed on a treadmill mounted into a metabolic chamber connected through a tube to an air pump used to maintain airflow inside the chamber (3.500 mL·min^−1^) and gas analysis was performed using an oxygen and carbon oxide analyzer (Sable Systems SS3, FC-10a O_2_/CO_2_ analyzer, NV, USA). Peak VO_2_ was calculated using the measured flow through the metabolic chamber, the expired fraction of effluent oxygen, and the fraction of oxygen in room air.

### 2.4. Cardiovascular Measurements and Myocardial Contractility

Heart rate and blood pressure were determined noninvasively using a computerized tail-cuff system (BP-2000, Visitech Systems) described elsewhere [19]. Rats were acclimatized to the apparatus during daily sessions over 4 days, one week before starting the experimental period. Heart rate and blood pressure were obtained before and after swimming training protocol.

Left ventricular function was measured as left ventricular (LV) *dP*/*dt*_max⁡_ and LV *dP*/*dt*_min⁡_, which are, respectively, the maximum and minimum rate of LV pressure increase. Briefly, rats were anesthetized with pentobarbital (0.1 mL/100 mg) and a polyethylene catheter was inserted in the right carotid artery and then positioned into the LV. LV pressure was determined by detecting the inflection point in the wave trace of LV diastolic pressure via a pressure transducer (YS100, Transonic System Inc., NY, USA). The analysis was performed using an analog-to-digital interface (Dataq Instruments, Akron, OH, USA). This program allows the derivation of LV wave pressure and the detection of maxima and minima of these curves beat-to-beat, providing the derived values of contraction (*dP*/*dt*_max⁡_) and relaxation (*dP*/*dt*_min⁡_). Values were expressed as mmHg·s^−1^.

### 2.5. Cardiac Structural Analysis

Twenty-four hours after the last exercise training session, all rats were killed and their tissues were harvested. A subset of hearts was stopped in diastole (14 mM KCl) and dissected to obtain the cardiac chambers. Paraffin-embedded cardiac sections of the LV were dewaxed using series of xylene and ethanol and further rehydrated. Then, these sections were stained with hematoxylin and eosin (H&E) for examination by light microscopy. Cardiomyocytes with visible nucleus and intact cellular membranes were included in the analysis. Cardiac myocyte was measured in the LV free wall with a computer-assisted morphometric system (Leica Quantimet 500, Cambridge, UK). For each animal approximately 15 visual fields were analyzed.

### 2.6. Lipid Peroxidation

The ferrous oxidation-xylenol (FOX) orange assay [20] was adapted for quantifying lipid hydroperoxides in heart extracts. Briefly, cardiac samples were homogenized (1 : 4 wt/vol) in phosphate buffer (50 mM, pH 7.8) and centrifuged at 12,000 g for 15 min at 4°C. 250 *μ*g of protein was mixed with 200 *μ*L FOX reagent containing 250 *μ*M ammonium ferrous sulfate, 100 *μ*M xylenol orange, and 25 mM H_2_SO_4_ and incubated at room temperature for 30 min. Absorbance of samples was read at 560 nm and the hydroperoxide concentration was calculated from the difference of the absorbance of the blank and tested samples.

### 2.7. Immunoblotting

Cardiac extracts from control, SHR, and SHRt animals were loaded into polyacrylamide gels (10%), submitted to electrophoresis, and proteins were electrotransferred to nitrocellulose membrane (BioRad Biosciences; Piscataway, NJ, USA). 0.5% Ponceau S staining was used to monitor equal loading of samples and transfer efficiency to the blot membrane. The blotted membrane was then blocked (5% nonfat dry milk, 10 mM Tris-HCl (pH = 7.6), 150 mM NaCl, and 0.1% Tween 20) for 2 h at room temperature and then incubated overnight at 4°C with specific antibodies against 4-HNE-protein adducts (Millipore, MA, USA), polyubiquitinated proteins, and 20S proteasome (*α*5/*α*7, *β*1, *β*5, and *β*7 subunits) (Biomol Int., PA, USA), Atrogin (Abcam, Cambridge, UK), ALDH2 (Santa Cruz Biotechnology, CA, USA), *αβ*-crystallin and HSP25 (Stressgen, MI, USA), Bad (Santa Cruz Bio Inc., CA, USA), and MuRF-1, Bcl-2, and p-Bad_ser112_ (Cell Signaling Tech., MA, USA). Binding of the primary antibody was detected with the use of horseradish peroxidase- (HRP-) conjugated secondary antibody from the same host as primary for 2 h at room temperature and developed using enhanced chemiluminescence (Amersham Biosciences, NJ, USA) detected by autoradiography. Quantification analysis of blots was performed with the use of Scion Image software (Scion based on NIH image). Samples were normalized to relative changes in GAPDH (Advanced Immunochemical Inc., CA, USA) or Ponceau staining and expressed as percent of control.

### 2.8. Protein Carbonyl Levels

Protein carbonyl levels were determined as previously described [16]. The carbonyl groups in the protein side chains were derivatized to 2,4-dinitrophenylhydrazone (DNP hydrazone) by reacting with 2,4-dinitrophenylhydrazine (DNPH). The DNP-derivatized protein samples were separated by polyacrylamide gel electrophoresis followed by immunoblotting.

### 2.9. Proteasome Activity

Chymotrypsin-like activity of the proteasome was assayed using the fluorogenic peptide Suc-Leu-Leu-Val-Tyr-7-amido-4-methylcoumarin (LLVY-MCA, Biomol Int., PA, USA). Assay was carried out in a microtiter plate by diluting 25 *μ*g of cytosolic protein into 200 *μ*L of 25 mM Tris-HCl, pH 7.4, containing 25 *μ*M LLVY-MCA, 25 *μ*M ATP, and 5.0 mM Mg^2+^. The rate of fluorescent product formation was measured with excitation and emission wavelengths of 350 and 440 nm, respectively. Peptidase activity was measured in the absence and presence of the proteasome inhibitor epoxomicin (1 *μ*M) and the difference between the two rates was attributed to the proteasome. Proteasome activity was linear for 30 min under the conditions of the assays.

### 2.10. Real-Time RT-PCR

RNA was isolated from heart tissue with Trizol reagent (GIBCO Invitrogen). RNA concentration and integrity were assessed; cDNA was synthesized using Superscript III RNase H-Reverse Transcriptase (Invitrogen) at 42°C for 50 min and Real-Time RT-PCR was performed. The primers used for gene amplification are listed as follows.

1. Atrogin/MAFbx sense, 5′-TACTAAGGAGCGCCATGGATACT-3′;
2. Atrogin/MAFbx antisense, 5′-GTTGAATCTTCTGGAATCCAGGAT-3′.
3. MuRF-1 sense, 5′-GTGTGAGGTGCCTACTTGCT-3′;
4. MuRF-1 antisense, 5′-ACTCAGCTCCTCCTTCACCT-3′.
5. Cyclophilin sense, 5′AATGCTGGACCAAACACAAA3′;
6. Cyclophilin antisense, 5′-CCTTCTTTCACCTTCCCAAA-3′.

Real-Time RT-PCR for Atrogin, MuRF-1, and cyclophilin (housekeeping) genes were run separately and amplifications were performed with an* ABI Prism 7500 Sequence Detection System* by using* SYBR Green PCR Master Mix* (Applied Biosystems, CA, USA). The results were quantified as Ct values, where Ct is defined as the threshold cycle of the PCR at which the amplified product is first detected. Expression was normalized with cyclophilin levels as an endogenous reference.

### 2.11. Aldehyde Dehydrogenase 2 Activity

Enzymatic activity of ALDH2 was determined by measuring the conversion of NAD+ to NADH, as described elsewhere [21]. The assays were carried out at 25°C in 50 mM sodium pyrophosphate buffer (pH 9.5) in the presence of 300 *μ*M acetaldehyde. Measurement of ALDH2 activity in the rat myocardium was determined by directly adding 80 *μ*g of the total lysate of the myocardium to the reaction mix and reading absorbance at 340 nm for 10 min. The empirical formula to calculate ALDH2 activity in units of *μ*mol NADH formed per minute per mg protein was *A* = *S* × 1000/(6.22 × 0.08), where 6.22 is the millimolar extinction coefficient of NADH at 340 nm and 0.08 is the target protein mass (mg) in the assay.

### 2.12. Statistical Analysis

Data are presented as means ± standard error of the mean (SEM). Data normality was assessed through Shapiro-Wilk's test. Two-way analysis of variance (ANOVA) for repeated measures with a* post hoc* testing by Tukey was used to compare the effect of exercise training on aerobic capacity (distance run and VO_2max⁡_), body weight, heart weight, and hemodynamic measurements. One-way ANOVA with a* post hoc* testing by Tukey was used to analyze cardiac hypertrophy, lipid peroxidation, protein expression, proteasome activity, ALDH2 activity, and mRNA levels in SHR compensated cardiac hypertrophy animal model. Statistical significance was considered to be achieved when the value of *p* was <0.05.

## 3. Results

### 3.1. Swimming Training Decreases Systolic Blood Pressure and Improves Aerobic Capacity in Hypertension

**Figure 1:**
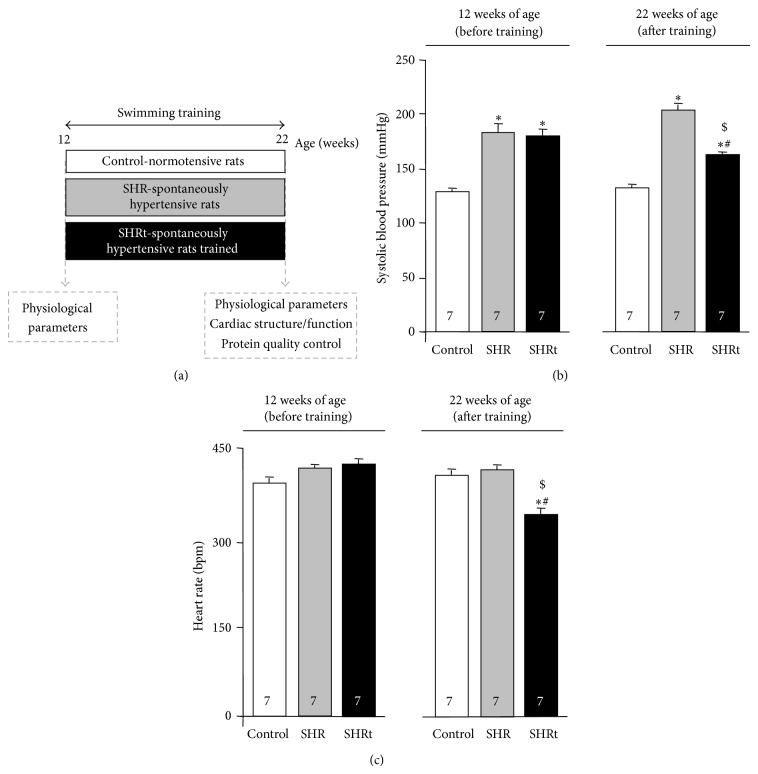
Exercise training reduces blood pressure and promotes bradycardia in hypertensive rats. Study design (a). Systolic blood pressure (b) and heart rate (c) in control (normotensive), sedentary spontaneously hypertensive rats (SHR), and swimming-trained SHR (SHRt) before and after 10 weeks of experimental protocol. Data are presented as mean ± SEM. ^∗^ *p* < 0.05 versus control. ^#^ *p* < 0.05 versus SHR. ^$^ *p* < 0.05 versus before swimming training period.

At twelve weeks of age (before swimming training, Figure 1(a)), SHR displayed high blood pressure (Figure 1(b)) with no changes in baseline heart rate (Figure 1(c)), exercise capacity, oxygen uptake, and body weight (Table 1) compared to age-matched control animals. These results corroborate our previous findings [15].

**Figure 2:**
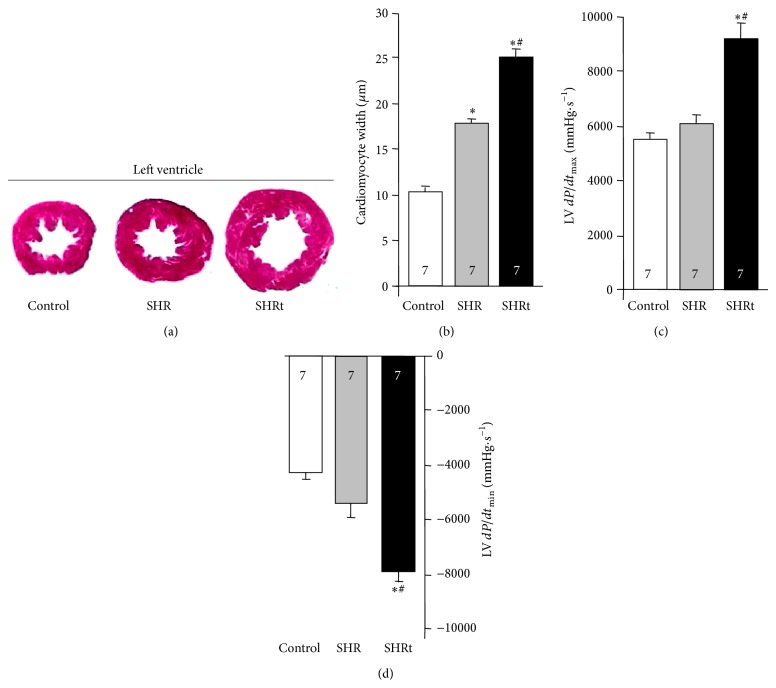
Exercise training improves myocardial contractility in hypertensive rats. Representative images of left ventricle (LV) (a). Cardiomyocyte width (b) and peak positive (LV *dP*/*dt*_max⁡_) (c) and peak negative (LV *dP*/*dt*_min⁡_) (d) values of the first derivative of the LV pressure in control (normotensive), sedentary spontaneously hypertensive rats (SHR), and swimming-trained SHR (SHRt). Data are presented as mean ± SEM. ^∗^ *p* < 0.05 versus control. ^#^ *p* < 0.05 versus SHR.

At the end of the experimental protocol, both control and hypertensive rats (twenty-two weeks of age, Figure 1(a)) reduced peak oxygen uptake by 35% and increased body weight compared to before experimental protocol period (week 12, Table 1). SHR developed cardiac hypertrophy compared to normotensive rats (Figure 2(a)), depicted by increased left ventricle : body weight ratio (Table 1) and augmented cardiomyocyte width (Figure 2(b)). Ten weeks of exercise training significantly improved exercise capacity in SHR group by increasing both running capacity and peak oxygen uptake (Table 1). The increased aerobic capacity seen in exercised SHR was paralleled by diminished blood pressure (Figure 1(b)) and resting bradycardia (Figure 1(c)). Interestingly, these changes were accompanied by a further increase in cardiac hypertrophy, characterized by higher left ventricle : body weight ratio (Table 1) and cardiomyocyte hypertrophy (Figures 2(a) and 2(b)). These findings demonstrate that aerobic exercise training improves hemodynamic parameters and cardiac hypertrophy in SHR, corroborating our previous findings [17].

### 3.2. Swimming Training Counteracts Cardiac Oxidative Stress and Improves Myocardial Function in Hypertension

**Figure 3:**
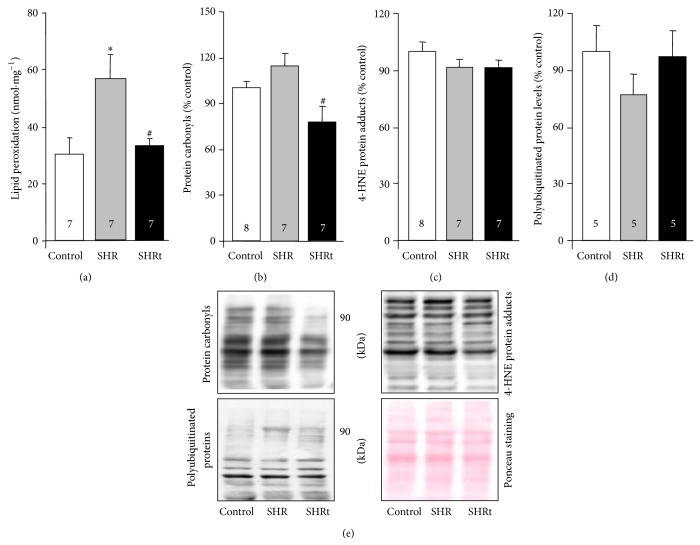
Swimming training decreases cardiac oxidative stress during compensated hypertrophy. Lipid peroxidation (a), protein carbonyls (b), 4-HNE-protein adducts (c), polyubiquitinated proteins (d), and their representative blots (e) in heart lysate from control (normotensive), sedentary spontaneously hypertensive rats (SHR), and swimming-trained SHR (SHRt). Protein expression was normalized by Ponceau staining. Data are presented as mean ± SEM. ^∗^ *p* < 0.05 versus control. ^#^ *p* < 0.05 versus SHR.

Increased oxidative stress has been shown to contribute to cardiac remodeling and dysfunction in a number of cardiovascular diseases [8, 9, 16, 22]. In order to evaluate the cardiac redox status during compensated hypertrophy in hypertension, we first measured ventricular lipid hydroperoxides in SHR and control groups. Our data demonstrate that there is a significant increase in cardiac lipid hydroperoxides in SHR compared to normotensive rats (Figure 3(a)).

Since cardiac oxidative stress precedes ventricular dysfunction in hypertension, we decide to measure myocardial contractility in SHR. No changes were observed in both maximum (LV *dP*/*dt*_max⁡_) and minimum (LV *dP*/*dt*_min⁡_) rate of left ventricle pressure peak in SHR when compared to control group (Figures 2(c) and 2(d)). These data reinforce our previous findings showing that twenty-two-week-old SHR present a compensated cardiac hypertrophy [23–25].

We next set out experiments to better understand the protective mechanisms evolved during compensated cardiac hypertrophy in hypertension. Considering that during lipid peroxidation free radicals attack polyunsaturated fatty acids and produce other cytotoxic compounds that react with proteins and DNA [26], we decided to measure well-known protein modifications caused by excessive lipid peroxidation such as protein carbonyls and 4-HNE-protein adducts. SHR did not present altered levels of protein carbonyls, 4-HNE-protein adducts, and polyubiquitinated proteins compared to age-matched normotensive rats (Figures 3(b)–3(e)).

Ten weeks of swimming training reduced LV lipid peroxidation and improved myocardial function by 40% in SHR compared to age-matched sedentary rats (Figures 2(c) and 3(a)). Moreover, exercise training reduced cardiac protein carbonyls without affecting 4-HNE-protein adducts and polyubiquitinated protein levels in SHR (Figures 3(b)–3(e)).

### 3.3. Hypertensive Rats Present Increased Cardiac Activity of Proteasome and Aldehyde Dehydrogenase 2 during Compensated Hypertrophy

In order to characterize the cardiac protein quality control profile during hypertension-induced compensated hypertrophy we evaluated E3 ubiquitin ligases Atrogin and MuRF-1, proteasomal *α*5/*α*7, *β*1, *β*5, and *β*7 subunits, and ALDH2 protein levels in heart homogenates from control, SHR, and SHRt. We also measured the catalytic activity of ALDH2 and proteasome (main players in the clearance of reactive aldehydes generated during lipid peroxidation and damaged proteins, resp.).

**Figure 4:**
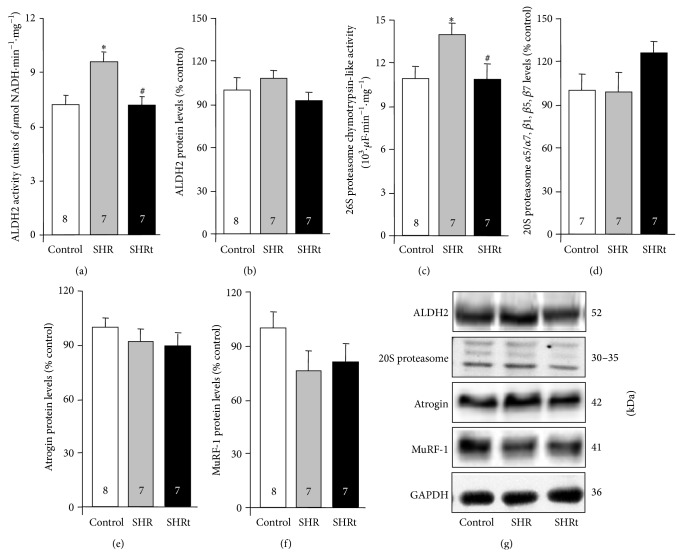
Hypertensive rats present increased cardiac activity of aldehyde dehydrogenase 2 and proteasome during compensated hypertrophy. Aldehyde dehydrogenase 2 (ALDH2) activity (a), ALDH2 protein levels (b), proteasome chymotrypsin-like activity (c), 20S proteasome *α*5/*α*7, *β*1, *β*5, and *β*7 protein levels (d), and E3 ubiquitin ligases Atrogin (e) and MuRF-1 (f) protein levels and their representative blots (g) in heart lysate from control (normotensive), sedentary spontaneously hypertensive rats (SHR), and swimming-trained SHR (SHRt). Protein expression was normalized by GAPDH. Data are presented as mean ± SEM. ^∗^ *p* < 0.05 versus control. ^#^ *p* < 0.05 versus SHR.

SHR displayed increased cardiac ALDH2 activity compared to normotensive rats (Figure 4(a)). SHR did not affect ALDH2 protein levels (Figure 4(b)). SHR presented a significant increase in 26S proteasome activity (Figure 4(c)) with no changes in 20S proteasome levels compared to control (Figure 4(d)). We also observed a 4-fold increase in Atrogin mRNA levels during compensated cardiac hypertrophy in SHR compared to age-matched control rats (437 ± 59 versus 100 ± 20, % of control). However, no changes in Atrogin protein levels were observed (Figure 4(e)). MuRF-1 mRNA and protein levels were not different between groups (Figure 4(f)). Ten weeks of swimming training reduced both ALDH2 and 26S proteasomal activity (Figures 4(a) and 4(c)) in SHR toward control values without affecting E3 ubiquitin ligase levels (Figures 4(e) and 4(f)).

**Figure 5:**
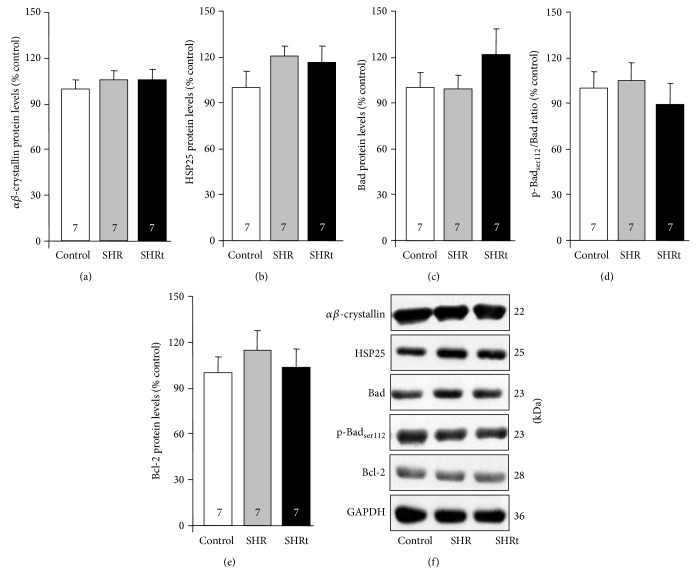
Cardiac levels of small chaperones and apoptosis-related markers are unchanged in compensated hypertrophy in hypertensive rats. Protein levels of small chaperones *αβ*-crystallin (a) and HSP25 (b), and apoptosis-related markers Bad (c), phospho Bad at serine 112 (d) and Bcl-2 (e), and their representative blots (f) in heart lysate from control (normotensive), sedentary spontaneously hypertensive rats (SHR), and swimming-trained SHR (SHRt). Protein expression was normalized by GAPDH. Data are presented as mean ± SEM.

In order to characterize the cardiac protein quality control in SHR we measured protein levels of small chaperones *αβ*-crystallin and HSP25 as well as apoptosis-related markers. Cardiac *αβ*-crystallin and HSP25 protein levels did not change in SHR compared to control rats (Figures 5(a) and 5(b)). No changes in cardiac proapoptotic Bad and p-Bad_ser112_ and antiapoptotic Bcl-2 protein levels were found in SHR compared to normotensive rats (Figures 5(c)–5(e)). Exercise training had no effect on cardiac small chaperones and apoptosis-related markers in SHR (Figures 5(a)–5(e)).

## 4. Discussion

Elevated blood pressure directly contributes to the development of LV hypertrophy, an important risk factor for morbidity and mortality [27]. The hypertension pathophysiology is characterized by a compensatory LV hypertrophy (usually at early stages) followed by a pathological cardiac remodeling depicted by increased cardiac mass, fibrosis, microvascular rarefaction, apoptosis, fetal gene reprogramming, and cardiac dysfunction, which ultimately leads to heart failure [3]. Therefore, blocking the transition from compensated cardiac hypertrophy to decompensated ventricular remodeling is required to reduce the hypertension-related morbidity and mortality.

A number of studies have recently focused on identifying intracellular multieffector brakes to suppress or reverse LV remodeling, which would become attractive targets for hypertension therapy. In the present study we found that twenty-two-week-old SHR displayed cardiac hypertrophy with preserved ventricular function and increased cardiac lipid peroxidation compared to normotensive rats. Hypertensive rats also presented increased ALDH2 and 26S proteasome activity along with no changes in protein carbonyls, 4-HNE-protein adducts, polyubiquitinated proteins, small chaperones, and apoptosis-related markers. Exercise training exacerbated cardiac hypertrophy, improved ventricular function, induced resting bradycardia, and decreased blood pressure in SHR. These changes were accompanied by reduced oxidative stress and decreased ALDH2 and proteasomal activity without affecting small chaperones levels and apoptosis. Overall, our findings suggest a compensatory activation of cardiac ALDH2 and proteasome in order to maintain protein quality control during hypertension-induced compensatory cardiac remodeling. Moreover, exercise training minimizes ALDH2 and proteasome activation by reducing cardiac stress, demonstrated by better redox profile.

Activation of UPS (including overexpression of genes encoding ubiquitin ligases and proteasome subunits) has been described in different models of cardiac hypertrophy [28–31]. However the meaning of UPS activation in cardiac hypertrophy is still under debate. Some studies suggest that UPS activation plays a detrimental role in hypertrophy, since sustained pharmacological proteasomal inhibition reverses ventricular hypertrophy in a rodent model of cardiac overload [28, 30]. Other studies demonstrate that either genetic or pharmacological inactivation of UPS promotes maladaptive cardiac remodeling and contributes to the onset of heart failure due to elevated proteotoxicity [8, 31, 32].

We found that during compensated hypertrophy there is an activation of cardiac proteasome with no signs of proteotoxicity (depicted by unchanged levels of cardiac small chaperones, polyubiquitinated proteins, and protein carbonyls). Indeed, exercise training reduces proteasomal activity and increases cardiac hypertrophy, suggesting that elevated proteasomal activity is not necessarily associated with increased cardiac mass. We have previously demonstrated that exercise training reestablishes cardiac proteasomal activity and improves ventricular function in heart failure animals without changing cardiac mass [16].

Oxidative stress plays a key role in the pathophysiology of hypertension. Sustained accumulation of free radicals due to disrupted redox homeostasis negatively affects vascular function and cardiac remodeling, therefore contributing to the onset and progression of hypertension. LV pathological remodeling is clearly marked by increased oxidative stress [22].

Here, we described that SHR already present increased cardiac oxidative stress during compensated cardiac hypertrophy, depicted by accumulation of lipid peroxides. Of interest, our findings demonstrate that, at this stage, there is no accumulation of cardiac 4-HNE-protein adducts in SHR. 4-HNE is the major cytotoxic aldehyde generated during lipid peroxidation [33, 34]. Our data suggest that, during hypertension-induced compensated cardiac hypertrophy, the increased 4-HNE generation through lipid peroxidation is counteracted by elevated ALDH2 activity, the main enzyme responsible for removing intracellular 4-HNE. ALDH2 has recently emerged as a key enzyme in the maintenance of cardiac homeostasis, since it efficiently eliminates toxic aldehydes (i.e., 4-HNE and acetaldehyde) by catalyzing their oxidation to nonreactive acids [11, 35, 36].

Exercise training significantly reduced cardiac lipid peroxidation along with improved ventricular function in SHR. Exercise training has been widely recognized as an effective nonpharmacological strategy for preventing and treating hypertension [17, 22, 37–39]. These data highlight the benefits of physical activity in protecting against the establishment of pathological cardiac remodeling in hypertension. However, considering the high complexity of both protein quality control machinery and redox balance-related mechanisms, further investigations need to be conducted in order to establish a cause-and-effect relationship as well as clarify other possible mechanisms regulated by physical activity in hypertension.

**Figure 6:**
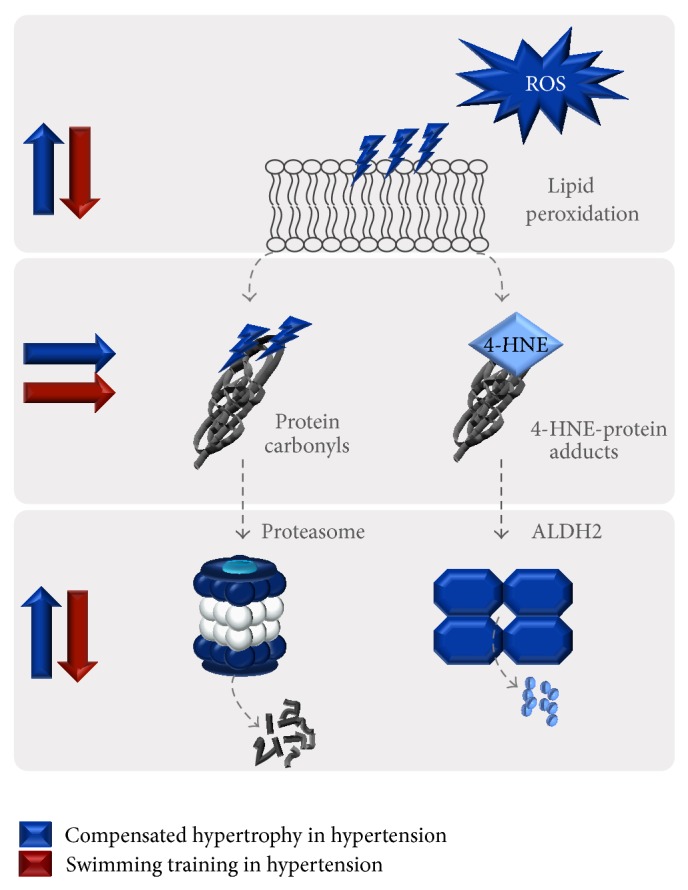
A proposed model for protein quality control during hypertension-induced compensatory cardiac hypertrophy: impact of swimming training. ROS, reactive oxygen species; 4-HNE, 4-hydroxynonenal; ALDH2, aldehyde dehydrogenase 2.

In summary, we provide evidence that hypertension-induced compensated cardiac hypertrophy is characterized by left ventricle oxidative stress. However, these changes are not followed by accumulation of cardiac protein carbonyls and 4-HNE-protein adducts. This scenario is explained, at least in part, by the increased catalytic activity of both ALDH2 and proteasome in SHR (main players in the clearance of reactive aldehydes generated during lipid peroxidation and damaged proteins, resp.). Ten weeks of swimming training improves ventricular function without affecting cardiac protein quality control in SHR (Figure 6). Altogether, our data highlight the importance of exercise training as a nonpharmacological intervention to prevent the progression of hypertension.

## Figures and Tables

**Table 1:** Physiological parameters.

Parameter	Before exercise training			After exercise training		
	Control	SHR	SHRt	Control	SHR	SHRt
Distance run (meters)	477 ± 15	510 ± 24	480 ± 12	430 ± 26	449 ± 29	745 ± 33^$∗#^
Peak VO_2_ (mL O_2_·kg^−1^·min^−1^)	67 ± 4	69 ± 2	70 ± 3	57 ± 3^$^	59 ± 2^$^	79 ± 2^$∗#^
BW (g)	256 ± 10	273 ± 07	275 ± 09	337 ± 9^$^	349 ± 6^$^	298 ± 8^∗#^
LVW : BW (%)	—	—	—	2.3 ± 0.1	2.8 ± 0.1^∗^	3.2 ± 0.1^∗#^

Distance run, peak oxygen uptake (VO_2_), and body weight (BW) data were obtained before and after ten weeks of experimental protocol from control (normotensive), sedentary spontaneously hypertensive rats (SHR), and swimming-trained SHR (SHRt) (*n* = 7 per group). Only left ventricle weight : BW ratio (LVW : BW) was obtained after the experimental protocol has ended. Data are presented as mean ± SEM. ^∗^ *p* < 0.05 versus control; ^#^ *p* < 0.05 versus SHR. ^$^ *p* < 0.05 versus before swimming training period.
